# Biomarkers in the Early Detection of Dementia: A Systematic Review

**DOI:** 10.7759/cureus.106323

**Published:** 2026-04-02

**Authors:** Emma Putnam, Sabine Katsoulos, Raymond L Ownby, Marc M Kesselman

**Affiliations:** 1 Medicine, Nova Southeastern University Dr. Kiran C. Patel College of Osteopathic Medicine, Fort Lauderdale, USA; 2 Neurology, Memorial Healthcare, Hollywood, USA; 3 Psychiatry and Behavioral Sciences, Nova Southeastern University Dr. Kiran C. Patel College of Osteopathic Medicine, Davie, USA; 4 Rheumatology, Nova Southeastern University Dr. Kiran C. Patel College of Osteopathic Medicine, Davie, USA

**Keywords:** alzheimer’s dementia, biomarkers, biosensor, cognitive impairment and dementia, screening and early detection

## Abstract

Early detection of Alzheimer’s disease (AD) and related dementias is critical for timely intervention and disease management. Mild cognitive impairment represents a transitional state between normal aging and dementia, but diagnosis remains challenging. Biomarkers offer promising tools for identifying early neurodegenerative changes, potentially years before clinical symptoms appear.

This systematic review examined studies published between 2012 and 2025 from PubMed and Ovid MEDLINE, focusing on the role of biomarkers in the early diagnosis of AD. Search terms included "biomarkers," "Alzheimer’s disease," "early diagnosis," and "early detection." Included studies were systematic reviews that had been peer-reviewed and emphasized early-stage diagnostic utility. Observational studies, meta-analyses, and systematic reviews were included and screened using a Preferred Reporting Items for Systematic Reviews and Meta-Analyses (PRISMA) flow diagram.

Biomarkers identified included cerebrospinal fluid (CSF) markers (amyloid beta, total tau, phosphorylated tau), genetic markers (ApoE4, presenilin mutations), and neuroimaging techniques (MRI, PET, fNIRS). Novel biomarkers such as miRNAs and biosensors, while still largely investigational, also show emerging potential. Phosphorylated tau, particularly p-tau 217, and the amyloid beta 42:40 ratio demonstrate high sensitivity in some studies for early AD pathology. In certain experimental studies, genetic and imaging biomarkers can detect risk or structural changes well before symptom onset.

While biomarkers cannot currently replace clinical diagnosis, they significantly enhance early detection and risk stratification. Further research is needed to establish standardized thresholds and to evaluate the ethical implications of widespread biomarker testing. Non-invasive, cost-effective tools such as CSF (minimally invasive) and plasma-based (non-invasive) assays and biosensors represent the future of early dementia diagnostics.

## Introduction and background

Early detection of dementia or pre-dementia states is crucial to prevent or slow the disease process. Early identification and quantification of cognitive changes in older individuals are essential for this purpose. It is important to note that dementia is not a single condition but a group of neurocognitive disorders. According to the DSM-5-TR, dementia is classified as a major neurocognitive disorder, which involves a significant and progressive decline in cognitive abilities that disrupts daily functioning [1]. The DSM-5 also emphasizes that cognitive deficits in both dementia and mild cognitive impairment (MCI) are not solely attributable to delirium or other mental disorders [1]. A key distinction is that in MCI, cognitive deficits do not impair the ability to function independently in daily life [1].

While dementia can arise from various underlying causes, with variable presentations, common diagnostic features include impairments in memory, language, mood, attention, and executive function, often reported by the patient or a knowledgeable informant [2]. Diagnosis typically involves a combination of history taking, neurological examination, cognitive testing, and imaging studies. Cognitive tests such as the Montreal Cognitive Assessment (MoCA) or the Mini-Mental State Examination (MMSE) are commonly used to assess cognitive function [2]. If necessary, further imaging studies, such as MRI, MRA, CTA, or EEG, may be used to help characterize the neurocognitive disorder. It is also important not to overlook potential metabolic, psychiatric, or infectious causes of symptoms. 

Biomarkers are a promising tool for diagnosing dementia, and they may eventually help detect the condition before symptoms emerge. Currently, biomarkers are primarily used in research settings within NIA-AA guidelines; they can be used in unique clinical scenarios. Mainly, if a clinician believes the information gathered from biomarkers will change disease management [3,4]. The amyloid-tau-neurodegeneration framework has been widely adopted in Alzheimer's dementia (AD) research to systematize biomarker classification and to delineate diagnostic criteria for MCI and dementia [4,5]. Biomarkers relevant to AD are typically categorized into two broad groups: biological markers and detection-based technologies. CSF biomarkers, genetic markers, and plasma biomarkers belong to the biological markers, while neuroimaging findings and miRNA/biosensors are detection-based technology [6-8]. Research primarily targets CSF amyloid beta and tau proteins, while imaging modalities such as MRI and PET detect amyloid deposition in the brain.

Amyloid beta is considered to be one of the more well-known biomarkers for AD detection. It is theorized that abnormal amyloid beta formation precedes all other neurodegenerative biomarkers in AD [9,10]. This is important, as amyloid beta fibrils are the main constituents of the plaques that form in the brain of AD patients [7]. The most accurate way to collect and determine the concentration of amyloid beta is through a lumbar puncture [7].

Post-translational modifications can lead to misfolded tau (phosphorylated tau), leading to aggregation of tau into oligomers and tangles, which is selectively increased in AD [7,10]. Oxidative stress, kinase activation, and impaired phosphatase activity all contribute to abnormal tau phosphorylation [10]. Tau can be phosphorylated and deposited in various regions of the brain, some of which correlate with disease severity [7]. Regional differences in tau deposition influence the primary presentation of dementia. For example, tau buildup in the posterior cortical area is associated with a visual-predominant variant of dementia [7]. It is also important to note the interplay between amyloid beta and tau pathology, as amyloid beta can enhance tau phosphorylation and aggregation, and vice versa [10].

Cortical tau deposition begins in the trans entorhinal cortex and spreads to other medial temporal lobes as the patient ages and the disease progresses [7]. Tau concentrated symmetrically in the temporoparietal region is most common in amnestic-predominant dementia. Asymmetric left temporal tau pathology primarily affects language, while widespread neocortical tau with sparing of the medial temporal lobes results in a dysexecutive-predominant variant of AD [7]. Additionally, once tau is phosphorylated, the misfolded protein can undergo cell-to-cell transmission, leading to further accumulation of misfolded tau [7].

There are four genetic markers commonly tested in patients with cognitive symptoms: Apolipoprotein E4 (ApoE4), amyloid precursor protein (APP), presenilin 1, and presenilin 2. These genetic markers can be ordered as risk stratification for development of MCI and do not currently have any diagnostic certainty. In individuals known to carry the ApoE4 allele or mutations in the presenilin genes, higher amyloid beta accumulation has been detected up to 22 years before the onset of dementia symptoms, impairments in glucose metabolism up to 18 years before symptoms appear, and cortical thinning up to 13 years prior [11]. The ApoE gene has multiple variants, but the E4 strain is associated with an increased risk of developing AD [12]. ApoE4 influences the seeding, aggregation, and clearance of amyloid beta proteins, and it binds to amyloid beta, promoting the formation of senile plaques [11]. 

Amyloid beta PET offers high sensitivity in detecting amyloid plaques in the brain and is currently considered the "gold standard" for amyloid plaque detection, although diagnostic criteria are always changing and adapting [13]. It is also a valuable tool for identifying individuals with MCI who are at increased risk of progressing to AD [7]. However, there is limited accessibility to this imaging modality due to the great expense [14].

MRI is a more accessible and cost-effective imaging modality used to assess structural brain changes in patients with cognitive impairments [13]. Brain MRI is considered a first-line neuroimaging tool for evaluating cognitive complaints, as recommended by guidelines from the American Academy of Neurology, the National Institute on Aging, and the American College of Radiology [5]. This is because MRI can help rule out structural causes of cognitive impairment, such as tumors, normal pressure hydrocephalus, or vascular contributions to dementia [5].

Functional near-infrared spectroscopy (fNIRS) is an emerging technology that can be used to characterize and aid in the diagnosis of MCI and dementia [3]. The spectrometer detects changes in hemoglobin oxygenation to assess brain activity [14]. One of the advantages of this technique is that it offers higher spatial resolution than EEG and higher temporal resolution than fMRI, with less sensitivity to motion artifacts [14]. However, it has limited clinical validation due to its novel status [14].

A newer biomarker target in dementia research is miRNA, which is easily detected in CSF or blood and exhibits good stability. This means miRNAs retain their structure and do not break down during sample preparation [12]. Studies by Doroszkiewicz et al. have found miRNAs to be involved in amyloid beta and tau signaling, inflammation, and apoptosis, all common pathologies underlying dementia [12]. The post-translational phosphorylation of tau protein is strongly linked to neurocognitive degeneration, and miRNAs that regulate this phosphorylation of the tau protein are being investigated as both potential biomarkers and therapeutic targets [12]. Additionally, there is evidence suggesting that miRNAs play a role in the regulation of APP and beta-secretase 1 (BACE1) expression, genes that increase amyloid beta production, and decrease the rate of degradation of amyloid beta plaques [12]. Experimental studies have shown promise in this area; however, there remain limitations with our ability to translate miRNA reliably [12].

Biosensors are an emerging tool for detecting the presence or absence of biomarkers in CSF or plasma samples in experimental studies. Because biomarkers often overlap across different dementia pathologies, using biosensors in a multi-biomarker strategy could improve the early diagnosis and treatment of affected individuals [6]. In the study by Bae et al., the biosensors used were primarily electrosensors, which contain a target analyte that binds to a transducer, generating a measurable electrical signal [6]. This study demonstrated the effectiveness of gold-based immunosensors, aptasensors, and molecularly imprinted polymers in detecting abnormal variants of amyloid beta, tau protein, and alpha-synuclein [6]. This data has been limited to experimental studies thus far, but shows promise for future research [12,13].

## Review

Methods

Search Strategy

A literature search was conducted to identify peer-reviewed articles related to biomarkers in the early recognition of Alzheimer's disease and dementia. Two primary electronic databases were utilized for the search: PubMed and Ovid Medline. The search was performed between 08/29/2025 and 09/30/2025, with the most recent search completed on 09/30/2025 and submission 01/2026. The following MeSH terms were used individually and in combination: “biomarkers”, “Alzheimer’s disease” AND “early diagnosis” AND “early detection” AND “dementia”. 

Inclusion and Exclusion Criteria

Articles were eligible for inclusion if they met the following criteria: Published in English, peer-reviewed original research or systematic reviews, focused on the use of biomarkers for the early detection or diagnosis of AD or related dementias, published between 2012 and 2025. It is important to note that both independent research studies and review articles were included, increasing the risk of overlap.

Articles were excluded if they met the following criteria: Non-peer-reviewed sources, editorials, letters, conference abstracts, or studies focusing solely on late-stage Alzheimer's disease, or articles unrelated to biomarkers or diagnostic approaches.

Screening and Selection

Search results from PubMed and Ovid were reviewed manually by one of the authors to remove duplicates. Titles and abstracts were screened for relevance based on the inclusion and exclusion criteria. Full texts of potentially relevant studies were retrieved and reviewed in detail. One of the authors reviewed studies using the 2024 Critical Appraisal Skills Program (CASP) for systematic reviews [15]. If the article in question met criteria in sections A-D, it was included. The PICO method was used for grouping and to narrow study selection. The population is patients at risk for dementia or cognitive impairment. The interest is in earlier and more specific diagnostic testing for cognitive impairment. The control in this study is the current gold standard of care for the diagnosis of cognitive impairment or dementia. The gold standard of care includes screening tools such as the MMSE or MOCA, imaging, including MRI and PET, and clinical signs and symptoms of cognitive impairment or dementia. The outcome studied was whether earlier testing with biomarkers impacts diagnostic accuracy or changes in the management of MCI (Table 1).

**Table 1:** General summary of the characteristics and findings of included studies. MCI: Mild cognitive impairment; AD: Alzheimer’s disease

Article Title	Summary
Diagnosis and management of dementia: a review	Review of the gold standard for diagnosis and management of dementia and pre-dementia states
Early screening model for mild cognitive impairment based on resting-state functional connectivity: A functional near-infrared spectroscopy study	Compared to healthy controls, patients with MCI showed significantly reduced connections in certain areas of the brain. Long range connections in prefrontal and occipital lobes have potential to be biomarkers
Cognitive Impairment in Older Adults: Epidemiology, Diagnosis, and Treatment	Reviews cognitive impairment testing, ATN classification of biomarkers. Overview of serum, CSF, and imaging-based biomarkers currently in clinical use.
The Value of Neuroimaging in Dementia Diagnosis	Discusses use and protocols for MRI and PET detection of biomarkers related to dementia
Recent advances in electrochemical biosensors for neurodegenerative disease biomarkers	Discusses use of biosensors for neurodegenerative disease biomarkers including alpha synuclein, amyloid beta and tau proteins
Mild cognitive impairment in clinical practice: A review article	Reviews background information on different types of MCI including etiology and screening tests
Biomarkers for neurodegenerative diseases	General overview of biomarkers, and future uses in treatment and early detection of various types of dementia
Biomarkers in subtypes of mild cognitive impairment and subjective cognitive decline	Discusses pathological biomarker burden correlating to severity or subtype of dementia
Phosphorylated Tau in Alzheimer’s disease and other Tauopathies	Background information specifically about Tau protein, especially in relation to development of cognitive impairment and dementia
Alzheimer’s disease: Insights and new prospects in disease pathophysiology, biomarkers and disease-modifying drugs	Discusses importance of early detection of dementia and how biomarkers could be the future of diagnosis and treatment
An exploration of distinguishing subjective cognitive decline and mild cognitive impairment based on resting-state prefrontal functional connectivity assessed by functional near-infrared spectroscopy	Use of fNIRS may help predict early stages of dementia
Molecular biomarkers and their implications for the early diagnosis of selected Neurodegenerative Diseases	Discusses molecular biomarkers and their implications for the early diagnosis of selected neurodegenerative disease, explores the role of miRNA in diagnostic testing
Comparative diagnostic performance of amyloid‐β positron emission tomography and magnetic resonance imaging in Alzheimer’s disease: A head‐to‐head meta‐analysis	Comparison of AB PET and MRI in diagnosis of AD
Cerebrospinal fluid levels of β-amyloid 1-42, but not of Tau, are fully changed already 5 to 10 years before the onset of Alzheimer's dementia	Discusses that CSF levels of beta amyloid 1-42 are fully changed 5-10 years before the onset of AD
Spatial patterns of neuroimaging biomarker change in individuals from families with autosomal dominant Alzheimer disease: a longitudinal study	Characterization of when and where biomarkers become abnormal along the disease course
An expanded role for neuroimaging in the evaluation of memory impairment	New strategies and neuroimaging for early diagnosis of dementia
Magnetic resonance imaging in Alzheimer’s disease and mild cognitive impairment	Discusses utility of biomarkers and imaging for improving diagnostic accuracy and earlier detection and management

Data Extraction and Synthesis 

**Figure 1:**
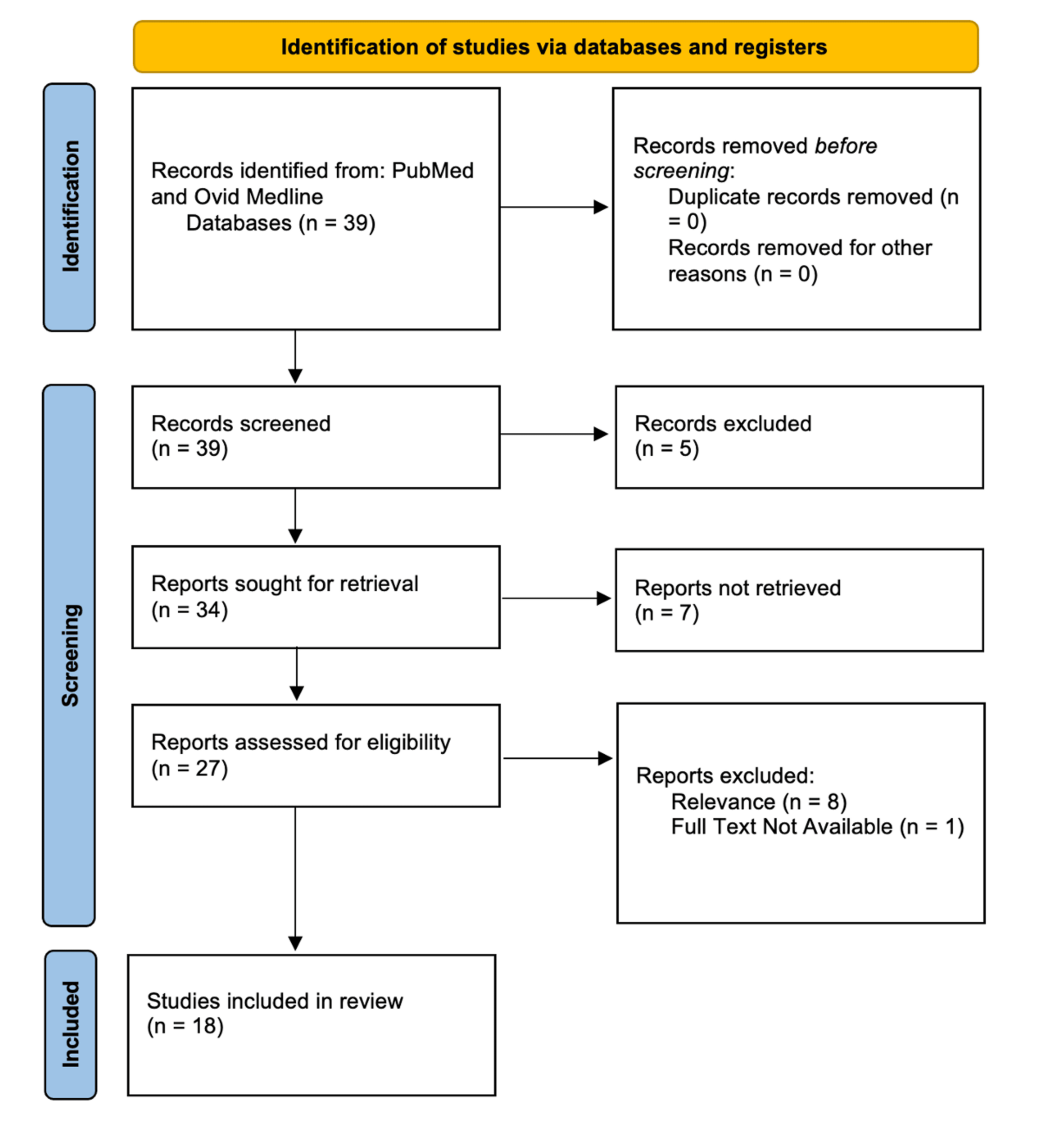
PRISMA 2020 flow diagram for a systematic review on biomarkers and early detection of dementia. The diagram illustrates the study selection process, from the initial identification of studies through electronic databases and other sources, screening of titles and abstracts, full-text screening, and final inclusion of studies in the systematic review. Reasons for exclusion at each stage are provided. PRISMA: Preferred Reporting Items for Systematic Reviews and Meta-Analyses

**Figure 2:**
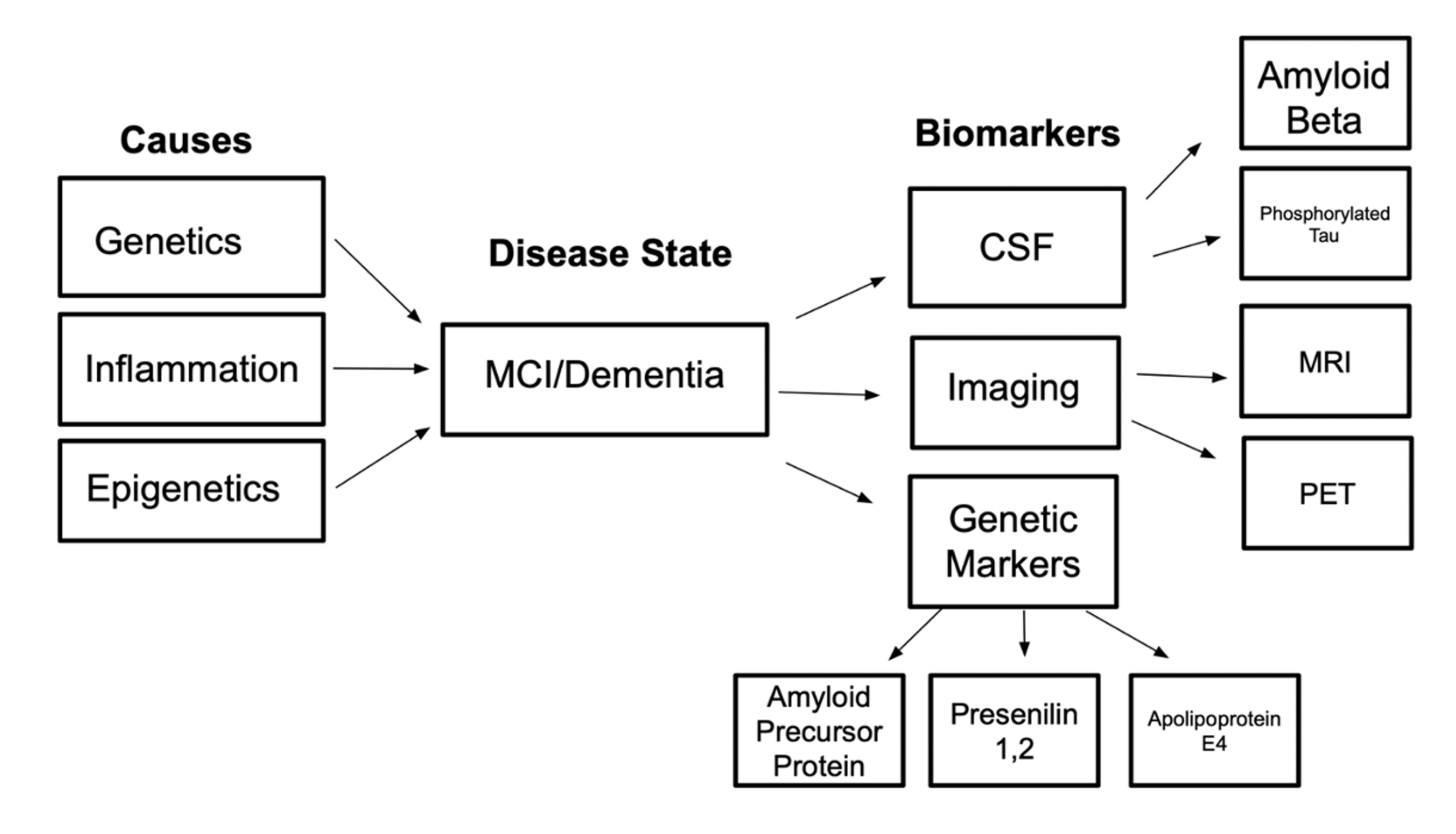
Visual representation of causes and measurable biomarkers associated with the disease states of MCI and dementia. MCI: Mild cognitive impairment

Data were extracted using a standardized procedure, using a form that captured the following elements: study design, sample size, type of biomarker assessed (e.g., CSF, imaging, blood-based, genetic), stage of disease at diagnosis, and key findings related to early detection. A narrative synthesis approach was used to analyze and summarize the findings, with attention to emerging trends, validated biomarkers, and gaps in the current literature (Figures 1, 2). Meta-analysis was not considered in this situation because the sources do not all use the same metrics for data collection.

Results

Biomarkers identified in this review include amyloid beta, phosphorylated tau, genetic markers, miRNA, and biosensors in addition to imaging studies including MRI, PET and fNIRS [3]. miRNA is a category of small noncoding RNA molecules that help to regulate gene functioning [13]. These small molecules have been implicated in many of the common etiologies behind cognitive impairment and dementia syndromes [13]. Biosensors are engineered devices to detect biomarkers including amyloid beta, phosphorylated tau and alpha synuclein [6]. They are a new and emerging technology that can accurately detect small amounts of target protein, which is promising for early detection and diagnosis of disease states (Table 2) [6].

**Table 2:** Summarized information about each biomarker, including advantages, disadvantages and early detection rates. Early detection rates are research findings and are not at this time a clinical guideline. fNIRS: Functional near-infrared spectroscopy

Biomarker	Advantages	Disadvantages	How Early Can We Detect
Amyloid Beta	Ratio of AB42:AB40 in CSF can be detected earlier than abnormal AB PET [7]; abnormal AB formation precedes other neurodegenerative biomarkers in AD [11]; very specific for AD	The main collection process is lumbar puncture, which is invasive [7]; not often performed outside of research settings [7]	5-20 years before symptom onset [16,17]
Phosphorylated Tau	Reliable assays have been developed for plasma and CSF [7]; diagnostic algorithms have been used for the prognosis of MCI patients [7]	Can be indicative of many neurodegenerative diseases	15-20 years [7]
MRI	Most cost-effective imaging technique [14]; can easily rule out other neurodegenerative processes [5]; many variations of MRI [18,19]; no radiation exposure	Only able to detect structural abnormalities [5,17]	Difficult to know when changes start, since imaging is not usually done without symptoms
PET	Highly sensitive for AB [10]	Expensive and not widely available [10]	Difficult to know when changes start, since imaging is not usually done without symptoms
Genetic Markers	Good for early detection of increased risk for developing dementia [11]	Knowing about increased risk for dementia may cause mental stress	13- 22 years before onset of symptoms [11]
fNIRS	Higher spatial resolution than EEG [12]; higher temporal resolution than fMRI with lower sensitivity to artifact [12]	Not widely available outside of research centers [12]	Not enough information available
miRNA	Good stability in both blood and CSF samples [8]; highly accurate in comparison to standardized methods [8]	Not widely available [8]; need to do more research into suitable target miRNAs	1-5 years prior to onset of symptoms [8]
Biosensors	Can be very specific to disease process [6]; can be useful for early detection and diagnosis [6]; could be useful in targeted treatment therapy [6]	More research needs to be done into sensor availability and accuracy [6]; not widely available [6]	Not enough available information

Discussion

Dementia cannot currently be diagnosed solely through biomarkers. A diagnosis typically requires consideration of clinical findings and cognitive testing in addition to biomarker data. However, biomarkers hold promise for early detection and prediction of cognitive impairment, as some research findings show changes in protein configurations can occur as early as 20 years before the onset of symptoms [7,16,17]. Furthermore, depending on the type of biomarker, researchers can classify MCI as high, intermediate, or low risk for progressing to AD [10]. This does not have implications for clinically accepted standards, only a framework for researchers. Risk is considered low if the patient has neurocognitive complaints but no evidence of biomarkers in the CSF [10]. For intermediate risk, there are neurocognitive complaints along with either positive AB with an untested other marker (i.e., phosphorylated tau) or untested AB with positive other markers [10]. It is important to note that within intermediate risk if AB is negative or not detected the risk is specific only to the potential development of AD; patients in this category are still at increased risk for other forms of dementia, such as frontotemporal dementia, vascular dementia, or dementia with Lewy bodies [10]. The risk of developing AD is considered high if patients exhibit neurocognitive complaints, AB42 pathology, and another biomarker indicative of neurodegeneration [10].

Regarding screening, further testing is recommended if cognitive symptoms begin before the age of 65, if symptom onset is rapid, or if there is impairment across multiple cognitive domains, but not specifically episodic memory [2]. The current screening age is set at 65 years due to the potential ethical, social, and psychological implications associated with earlier diagnosis. Identifying individuals at risk at a younger age may require patients to live with this knowledge for a prolonged period, despite the current limitations in effective treatments for cognitive impairment or dementia. This awareness may cause significant psychological distress, as individuals with an elevated risk may begin to interpret normal memory lapses as potential early indicators of dementia. Furthermore, there are important ethical considerations in disclosing to patients that they may develop a debilitating condition when the timing, certainty, and availability of effective treatment remain unclear.

CSF Biomarkers

Amyloid beta: The measured ratio of AB42 to soluble AB40 aggregation in the CSF is often used to determine levels with accuracy highly positively correlated to AB PET [11]. Lumbar puncture is considered to be a minimally invasive test, as well as not widely available outside of specialist centers [8]. For these reasons, it is not often performed to determine AD. There have been some new investigations for plasma-based methods to determine amyloid beta load, including mass spectrometry [7]. However, levels of plasma amyloid beta are even more decreased in comparison to levels in the CSF as it can be affected by production outside of the brain [7]. Additionally, these plasma-based and mass spectrometry-based methods are still considered to be investigational and not found to be as reliable as the standard techniques, however research is evolving in this field. 

Tau/Phosphorylated Tau: Phosphorylated tau 181 and 217 are likely to increase 15-20 years before the onset of symptoms, making them potentially useful for early detection and diagnosis of cognitive impairment [7]. Phosphorylated tau 217 in certain studies shows a stronger association with increased tau tangle load and disease severity than phosphorylated tau 181 and may also be able to distinguish AD from other dementias with higher accuracy than other isomers [7].

Similar to amyloid beta, tau is most often found in the CSF, but recently, more reliable assays have also been developed to detect phosphorylated tau in blood, with studies showing equivalent accuracy to CSF biomarkers [7]. Phosphorylated tau 181 plasma levels have been shown to correlate strongly with levels found in CSF and on PET scans [11]. High levels of phosphorylated tau in patients with MCI have been linked to an increased likelihood of developing AD, and diagnostic algorithms have been developed to predict prognosis [7].

Genetic Markers

ApoE4, Presenilin 1 and 2, amyloid precursor protein: ApoE is primarily implicated in cholesterol homeostasis; its different isoforms have varying functions. Abnormally folded ApoE4 can disrupt the distribution of cholesterol and other lipids, thereby reducing neuronal plasticity [11].

Mutations that increase amyloid precursor protein cleavage led to elevated amyloid beta levels, further increasing the risk of AD development. The presenilin 1 gene plays a crucial role in APP cleavage, which contributes to the accumulation of amyloid beta [13]. Studies have found disruptions in these genes to indicate higher probability of developing neurocognitive disease; however, they are not diagnostic and do not immediately imply the development of disease state.

Imaging Studies

MRI: The identification and exclusion of potentially reversible causes of cognitive impairment are essential to provide patients with the best possible care. One important characteristic of neurodegenerative diseases is that brain atrophy is almost always progressive and can have a variable presentation with each individual [17]. Therefore, even in “healthy” controls, the presence of progressive brain atrophy over time should raise clinical suspicion for the future development of a neurodegenerative condition [17].

Volumetric MRI (vMRI): vMRI is a specialized imaging technique that assesses morphological changes across the entire brain, with particular focus on medial temporal lobe volumes [11]. It enables the segmentation of the brain into neuroanatomic regions and quantifies tissue loss in each region [11,18]. 

Functional MRI (fMRI): fMRI, another variant of MRI, measures changes in blood flow and volume in different brain regions by capturing dynamic representations of brain activity using blood-oxygen-level-dependent (BOLD) signals [19]. Researchers have used fMRI to gain insight into the functional connectivity of intrinsic networks, including the default mode network (DMN) [19]. Chandra et al. reviewed multiple studies showing that, in early AD, there is decreased activity in the posterior DMN regions and increased activity in the anterior and ventral regions. However, between two to four years after symptom onset, a marked decrease in connectivity is observed across all DMN regions. Additionally, patients with MCI exhibit similar hippocampal deactivation during memory recall as those with AD. Similar findings have been observed in domains such as working memory, visuospatial ability, attention, semantic knowledge, and motor performance in patients with MCI. These patterns may serve as important markers for identifying early or prodromal stages of neurodegenerative disease [19]. 

PET: In studies by Eliassen et al., it was found that fluorodeoxyglucose-PET (FDG-PET) metabolism and entorhinal cortex thickness were reduced in individuals with amnestic MCI and subjective cognitive decline, compared to healthy controls [9]. Regional hypometabolism is associated with disease progression, as it reflects synaptic and metabolic dysfunction, as well as neuronal cell loss [9].

In recent years, PET scans targeting tau have started to gain attention, showing great promise for monitoring disease progression and treatment response [5]. While amyloid PET remains more commonly used, both amyloid and tau PET scans are still cost-prohibitive for many individuals [5]. However, some limitations for PET scans are that they can be expensive and only available at certain locations, limiting the number of people able to access this technology. 

fNIRS: Investigational studies showed that a significant difference was found in resting-state prefrontal functional connectivity on fNIRS when compared to neuropsychological tests of the same patients [12]. For instance, individuals with early-stage cognitive impairment or MCI exhibited increased abnormal cross-hemisphere functional connections within the ipsilateral cerebral hemispheres [12]. While previous studies have shown some abnormalities in brain functional connections during dementia, this study significantly expanded the number of connections we now know to be affected [12].

Additionally, in these studies, when compared to healthy controls, patients with MCI showed significantly reduced connectivity in certain areas of the brain, particularly between the prefrontal and occipital lobes [3]. Measurements of long-range connections in both the prefrontal and occipital lobes could serve as biomarkers, as signal intensity decreases in patients with cognitive impairments [3]. Zhang et al. found that MCI reduces cortical blood flow and induces structural changes, impairing the collaboration between functional brain areas [3]. This is particularly problematic, as it creates a self-destructive loop in brain function.

Other

miRNA: Currently, there are no standard guidelines for diagnosing AD or any other neurocognitive disorder based solely on miRNA data, but several promising research avenues have emerged. These miRNAs would make excellent biomarkers due to their availability in blood samples, making the collection process non-invasive and less expensive. Moreover, they could provide valuable insights into the underlying pathophysiology of cognitive impairment. Preliminary findings by Doroszkiewicz et al. propose that abnormal miRNA expression can be detected in asymptomatic individuals, potentially allowing for earlier detection up to five years before the clinical diagnosis of MCI or dementia [12]. While further research is needed, the stability, early detection potential, and non-invasive collection method make miRNA a promising biomarker that could significantly impact the lives of individuals with MCI or dementia.

Biosensors

Biosensors are a highly promising area of research, especially with the ability to accurately detect small amounts of these abnormal proteins; there are still many variables to consider. Some technical barriers with biosensors are that the shape of the nanosensor appears to influence its sensitivity in detecting different proteins [6]. Furthermore, additional research is needed to improve the production efficiency and accuracy of these sensors so they can be more widely distributed [6]. Overall, biosensors, while in a highly experimental status, hold great promise as a tool for detecting early changes in MCI and dementia pathology.

Limitations

Some limitations of this study include variability in the patient population, the lack of standardized diagnostic criteria, and the use of only two search engines. The absence of standardized biomarker-based diagnostic criteria for dementia and MCI is a significant limitation, as different studies applied varying standards to determine who qualified as having MCI or dementia thus affecting who and what data was included.

The patient populations also varied considerably across studies, particularly regarding eligibility criteria. There remains variability in who should be screened and how early screening should occur, which contributes to inconsistent inclusion criteria. For example, some studies included only patients who had already been diagnosed with MCI or dementia, while others evaluated individuals before the onset of symptoms.

Additionally, having a single reviewer screening and limiting the search to only two search engines may have introduced bias and restricted the overall breadth and diversity of the data collected. The absence of a quantitative synthesis in this review can decrease the effect of power of the study.

## Conclusions

There is a pre-dementia state, initially referred to as subjective cognitive decline, followed by MCI. Creating standardized diagnostic criteria remains challenging, as there are limited established cutoff points, making it difficult to compare findings across the current literature. A key factor for developing standardized diagnostic criteria would be to establish clear diagnostic thresholds with further studies, including outcome-based validation or longitudinal studies.

Biomarkers have the potential to be a useful tool in diagnosing pre-dementia states, but it is important to consider the potential negative implications of having this knowledge. For instance, in certain studies, misfolded proteins can be detected up to 20 years before the onset of symptoms, which could cause anxiety regarding future diagnoses. In cases where medical management would not change based on screening results, it may be useful to consider not using this screening, particularly in younger populations, who would have to live with this knowledge for a long time. Additionally, there are potential concerns about the implications for insurance coverage. If insurance companies gain access to such data, they could use it to increase premiums for individuals based on their higher risk of developing dementia and its potential sequelae. Further research into the use of biomarkers as a risk score may enable clinicians to identify patients with memory concerns who could benefit from additional diagnostic evaluation, even prior to the onset of memory symptoms.
